# Epithelial-stromal crosstalk and fibrosis in eosinophilic esophagitis

**DOI:** 10.1007/s00535-018-1498-3

**Published:** 2018-08-12

**Authors:** Amanda B. Muir, Joshua X. Wang, Hiroshi Nakagawa

**Affiliations:** 1 Division of Pediatric Gastroenterology, Hepatology, and Nutrition, The Children’s Hospital of Philadelphia, Philadelphia, PA 19104-6160 USA; 2 Department of Pediatrics, Perelman School of Medicine, University of Pennsylvania, Philadelphia, PA 19104 USA; 3 Division of Gastroenterology, Department of Medicine, Perelman School of Medicine, University of Pennsylvania, 956 Biomedical Research Building, 421 Curie Boulevard, Philadelphia, PA 19104-6160 USA; 4 Abramson Cancer Center, University of Pennsylvania, Philadelphia, PA 19104 USA

**Keywords:** Eosinophilic esophagitis, Fibrosis, Interleukin-13, Microenvironment, Transforming growth factor-β

## Abstract

Eosinophilic esophagitis (EoE) is a food allergen-induced inflammatory disorder. EoE is increasingly recognized as a cause of swallowing dysfunction, food impaction and esophageal stricture. Inflammation of the esophageal mucosa involves immune cell infiltrate, reactive epithelial changes and fibroblast activation, culminating in robust tissue remodeling toward esophageal fibrosis characterized by excess collagen deposition in the subepithelial lamina propria. Fibrosis contributes to a unique mechanical property of the EoE-affected esophagus that is substantially stiffer than the normal esophagus. There is a great need to better understand the processes behind esophageal fibrosis in order to foster improved diagnostic tools and novel therapeutics for EoE-related esophageal fibrosis. In this review, we discuss the role of esophageal inflammatory microenvironment that promotes esophageal fibrosis, with specific emphasis upon cytokines-mediated functional epithelial-stromal interplays, recruitment and activation of a variety of effector cells, and tissue stiffness. We then explore the current state of clinical methodologies to detect and treat the EoE-related esophageal stricture.

## Introduction

Eosinophilic esophagitis (EoE) is an allergic chronic inflammatory disorder affecting ~ 4 in 10,000 individuals in the United States. EoE involves transmural esophageal inflammation and subepithelial fibrosis, leading to esophageal stricture, the most serious clinical consequence of epithelial and lamina propria tissue remodeling events in EoE [1–3]. Clinical manifestations include dysphagia and food impaction, which impact patients’ quality of life and health care costs [4, 5]. The natural history of the disease is unknown, but retrospective studies suggest that the most common clinical symptoms reflect the progression and worsening of untreated esophageal inflammation and fibrosis [6]. While the pathogenesis of EoE involves food antigen exposure, genetics, and eosinophil migration into the esophageal epithelium, it remains unclear how these interactions contribute to fibrosis. Dietary elimination and steroids remain the standard of care for EoE; however, 40–50% of EoE patients, despite age, are refractory to first line treatments [4].  Approximately, 10% of EoE patients show extremely narrow-caliber esophagus and are difficult to treat [7]. At the time of diagnosis up to 67% of adults and 16% of children already have fibrostenotic disease [8]; once there is fibrosis, decreasing inflammation alone may not provide symptomatic relief.

Food and environmental allergens trigger a diverse esophageal inflammatory response, leading to a pathologic cycle of tissue damage and repair. Active EoE is diagnosed by the presence of intramucosal eosinophilia (≥ 15/hpf peak eosinophil count) following administration of proton pump inhibitor (PPI) for 8 weeks to rule out PPI-responsive esophageal eosinophilia [9]. The role of eosinophils in EoE remains elusive. Active EoE features cytolytic degranulation of eosinophils to release their granule proteins in the esophageal epithelium that entrap bacteria and fungi [10]. Thus, eosinophils may have a host defense role in the context of impaired mucosal barrier functions in EoE; however, the pathogenic role of eosinophils has been implicated in inflammation-related fibrosis of a variety of organs including the heart, the airway, and the skin [11]. While eosinophils are the most noticeable infiltrating cell population, there is actually a mixed inflammatory cell infiltrate comprising mast cells, basophils and lymphocytes [12–14]. In a mouse model of EoE induced by ova albumin, basophils have been implicated as a major effector in EoE-related inflammation [13].

The vigorous inflammatory state and progressive tissue damage promote esophageal fibrosis. Esophageal fibrosis is defined as excessive extracellular matrix (ECM) deposition, most notably collagen fibers, in the esophageal lamina propria. Fibroblasts are the major effector cells in fibrosis. They become activated in the setting of injury to provide the ECM proteins needed for wound healing. ECM proteins serve as the scaffolding for re-epithelialization and wound closure. In EoE, fibroblasts residing in the subepithelial lamina propria express markers of activated myofibroblasts such as α-smooth muscle actin (α-SMA) under inflammatory conditions. Chronic inflammation leads to continuous fibroblast activation, proliferation, and survival with excessive secretion of ECM components, increasing esophageal stiffness [15, 16]. Tissue stiffness is associated with esophageal dysfunction as evident clinically by dysphagia, food impaction, and stricture. Thus, understanding the mechanisms underlying fibrosis in EoE and identifying novel pharmacologic targets aimed at decreasing tissue stiffness and matrix remodeling are paramount to improving patient outcomes.

In this review we seek to define how inflammation promotes esophageal fibrosis in EoE. We will examine the effects of inflammation and tissue stiffness on esophageal remodeling. We will discuss the chemical microenvironment mediated by inflammatory cytokines that facilitates the functional interplays between epithelial cells and stromal fibroblasts. Additionally, we will discuss the mechanical microenvironment associated with tissue stiffness. Finally, we will evaluate the effects of tissue remodeling clinically, looking at the evolution of esophageal fibrosis in EoE and the methods we use to detect it.

## Mechanisms of fibrosis in EoE

Fibroblasts, immune cells, epithelial cells and the interactions of these key players via secreted inflammatory cytokines are all likely responsible for fibrosis in EoE. In addition to the rich chemical changes, tissue stiffness contributes to the progression of fibrosis. Herein we outline the complex cellular interplays in the inflammatory milieu, the stiffness of the esophagus, and the mechanism by which these factors drive fibroblast activation.

### Th2 immunity and fibrosis in EoE

**Fig. 1:**
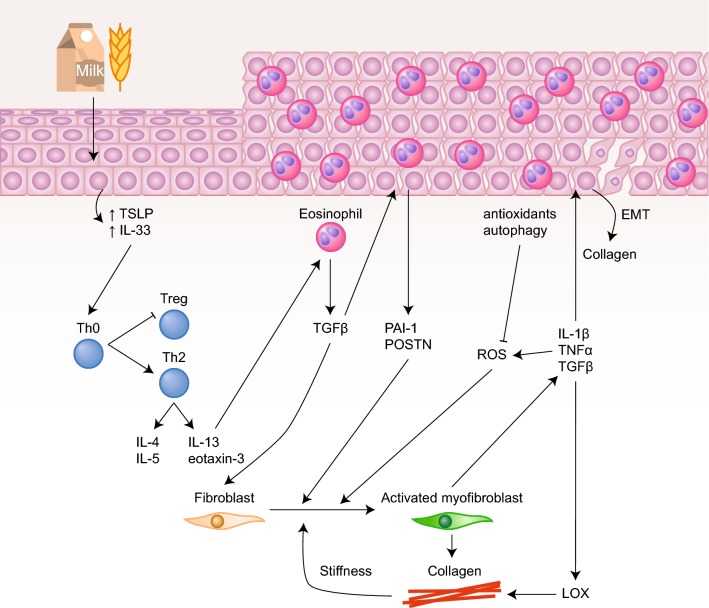
Food allergens trigger epithelial cell production of pro-inflammatory cytokines such as TSLP and IL-33 which drive T_h_2-predominant inflammation, recruiting eosinophils via T_h_2 cytokines such as IL-13, IL-5 and eotaxin3. These cytokines may stimulate esophageal epithelial cell proliferation while delaying terminal differentiation, resulting in basal cell hyperplasia (BCH). BCH may be associated with diminished epithelial characteristics via EMT with decreased epithelial barrier functions, leading to the aggravated inflammatory milieu. Multiple inflammatory cytokines trigger ROS production which is subjected to regulation by antioxidants and autophagy. ROS are also essential in EMT. Limited autophagy flux in the epithelial cells may promote EoE inflammation, BCH and fibrosis. EoE-relevant cytokines may recruit and stimulate fibroblasts to induce activated myofibroblasts. Activation of myofibroblasts also involves epithelial-stromal crosstalk mediated by cytokines and growth factors as well as tissue stiffness increased via collagen cross-linking

Multiple inflammatory cytokines play critical roles in EoE pathobiology. The inflammatory milieu in EoE is dominated by T helper-type 2 (T_h_2) lymphocytes characterized by production of interleukins (IL)-4, 5, and 13 [17]. Chronic T_h_2 inflammation leads to tissue fibrosis and end organ dysfunction [18, 19]. In EoE, it is believed that the food allergen-exposed esophageal epithelium releases cytokines thymic stromal lymphopoietin (TSLP) [20, 21] and IL-33 [22], potent enhancers of T_h_2-mediated immunity and trigger the inflammatory cascade (Fig. 1). Induction of T_h_2 inflammation leads to production of inflammatory cytokines IL-4, 5, and 13. As the major effector cytokine in EoE [23, 24], IL-13 stimulates epithelial production of eotaxin-3 (aka CCL26), a potent chemoattractant for eosinophils and basophils [25–27], and promote tissue eosinophilia [28, 29]. Additionally, tumor necrosis factor (TNF)-α, IL-4 and IL-13 act synergistically to induce eotaxin-3. The induction of eotaxin-3 occurs not only in the esophageal epithelium but also in esophageal fibroblasts via transcription factor STAT6 activated by T_h_2 cytokines [26, 30]. These cytokines cooperate to promote fibrosis via trans-differentiation of fibroblasts into activated myofibroblasts, the key effector cells in fibrosis [31, 32]. Additionally, fibroblasts are stimulated by eosinophil-derived factors such as transforming growth factor (TGF)-β and IL-1β [33]. Once activated, myofibroblasts secrete extracellular matrix components including collagen, proliferate, migrate, and become contractile. These abilities allow for normal wound healing in the setting of injury but in the pathologic state, robust and constant activation promote tissue stiffness, causing dysphagia, food impaction and esophageal stricture in EoE.

### IL-13 and fibrosis

Besides granulocyte recruitment, IL-13 mediates fibrotic tissue remodeling in murine models of fibrotic disorders. Treatment with anti-IL-13 (Tralokinumab) decreased airway fibrosis in mice [34]. In mice, lung-targeted transgenic IL-13 overexpression leads to epithelial hyperplasia, angiogenesis, eotaxin production, and subepithelial fibrosis with increased collagen deposition in the esophagus [35]. Furthermore, when IL-13 was overexpressed in an eosinophil deficient mouse, there was continued remodeling despite lack of eosinophilic infiltration [35], suggesting that IL-13 may drive esophageal fibrosis in a manner independent of its role in eosinophil recruitment. The mechanism by which IL-13 enhances fibroblast activation and collagen deposition in EoE is not completely understood. In a murine model of pulmonary fibrosis, IL-13 has been shown to induce fibroblast migration via IL-13-mediated enhanced formation of lamellipodia, cytoskeletal projections at the leading edge of the cells, as well as enhanced matrix metalloproteinases (MMPs) activity [36]. The role of IL-13 receptor (IL-13R)-mediated signaling has been tested in genetically engineered mice with defective IL-13Rα2, a decoy receptor competing with IL-13R for IL-13 [35], suggesting. Loss of *IL*-*13Rα2* resulted in enhanced IL-13R-mediated signaling and esophageal fibrosis in mice with transgenic IL-13 expression [35], suggesting a role for IL-13R in fibrotic tissue remodeling during the inflammatory process.

### TGFβ and fibrosis

The inflammatory infiltrate in EoE consists of eosinophils, mast cells and basophils. Activation of the T_h_2 responses leads to invasion of these granulocytes and robust production of the cytokine TGFβ leading to tissue damage and fibrotic tissue remodeling [12, 37]. We have previously shown that TGFβ stimulation of primary esophageal fibroblasts leads to enhanced expression of α-SMA, collagen and fibronectin [38]. Furthermore, esophageal biopsies of EoE patients demonstrate increased expression of TGFβ and phosphorylation of its downstream transcription factor SMAD2/3 compared with gastroesophageal reflux disease and normal control patients. There was co-localization of phosphorylated-SMAD2/3 with eosinophil granule proteins, suggesting that the eosinophils are the major source of TGFβ production [37].

A mouse model of EoE supports the role of canonical TGFβ signaling in EoE. In an ova albumin-induced model of murine EoE with impaired TGFβ signaling via Smad3 deficiency resulted in a decreased collagen deposition and angiogenesis, but not eosinophilia [31]. Thus, even with ongoing inflammation, inhibition of TGFβ leads to attenuated fibrosis. TGFβ also induces a number of other profibrotic tissue remodeling factors including MMPs, plasminogen activator inhibitor 1 (PAI-1) and Periostin. MMP2 and MMP14 are upregulated in EoE and are reduced in patients responding to corticosteroid therapy [39]. PAI-1 has also been found to be upregulated in active EoE patient biopsies and its expression correlates with lamina propria fibrosis. PAI-1 inhibition leads to diminished gene expression of profibrotic α-SMA [40]. Periostin is a glycoprotein expressed in both epithelial cells and fibroblasts that has been shown to be upregulated in the EoE transcriptome [41, 42]. In the setting of IL-4 and IL-13 stimulation, Periostin binds to integrins on the cell surface leading to fibroblast proliferation, activation, and production of collagen [43, 44]. However, IL-13 may activate tissue fibrosis in a TGFβ independent manner since IL-13 activated the fibrogenic machinery in mice with the impaired TGFβ signaling cascade [45]. Other TGFβ target genes essential in fibrosis and extracellular matrix remodeling, yet to be studied in the context of EoE, include connective tissue growth factor (CTGF) [46], insulin-like growth factor binding protein (IGFBP)-3 [47] and lysyl oxidase (LOX) [48]. In particular, LOX catalyzes collagen cross-linking. Besides, TGFβ stimulates smooth muscle hypertrophy, increasing tissue stiffness [12], which may increase with the EoE disease progression [49].

### Epithelial contributions to fibrosis in EoE

While the inciting events in EoE are unknown, damage to the epithelial barrier leading to T_h_2 immune response is likely an early initiator. EoE inflammation involves reactive epithelial changes leading to epithelial barrier defects [50]. The normal stratified squamous epithelium of the esophagus comprises a single layer of proliferative basal cells (keratinocytes) that exit cell division cycle in the suprabasal cell layers to undergo terminal differentiation and desquamate eventually into the esophageal lumen. This differentiation gradient is disrupted in EoE by basal cell hyperplasia (BCH) [51], an expansion of basaloid cells (> 20% of epithelial height) as well as edematous dilatation of the intercellular spaces (spongiosis), and the retention of nuclei in the superficial cell layer (parakeratosis) [52]. BCH contributes to barrier defect via downregulation of epithelial junction proteins. EoE-relevant cytokines promote epithelial barrier defects by downregulating desmoglein-1, which mediates cell–cell junction formation [41, 53]. In addition to its role in propagating inflammation, esophageal epithelial cells can act as effector cells in fibrosis. Conditioned media from esophageal epithelial cell culture stimulate esophageal fibroblasts to produce profibrotic cytokines IL-1β and TNF-α [32]. TNF-α and TGF-β stimulate epithelial-mesenchymal transition (EMT), by which epithelial cells take on characteristics of mesenchymal cells including collagen production, migration, and contraction [32, 54–56]. In normal esophageal keratinocytes, impaired squamous-cell differentiation triggers EMT [57]. BCH lesions express EMT markers in EoE [32, 54, 56]. Diminishing cell adhesion, EMT contributes to barrier defects [54, 58]. Additionally, keratinocytes that have undergone EMT display increased collagen production [56]. Thus, epithelial changes may influence the tissue microenvironment to facilitate fibrosis in a non-cell autonomous manner.

### Oxidative stress, redox homeostasis and fibrosis

Reactive oxygen species (ROS) are produced via cellular metabolism and are essential in physiological processes including cell signaling, proliferation, differentiation and metabolic adaptation [59, 60]. ROS modulate the balance between proliferation and differentiation in a variety of tissues types, including the esophagus [61]. EMT involves ROS and requires proper redox regulation in esophageal keratinocytes [62]. ROS have been implicated in immune cell mobilization and eosinophils generate ROS [63]. Multiple EoE-relevant cytokines including IL-5, IL-13, TNF-α and TGF-β induce ROS in esophageal keratinocytes [64]. While excessive ROS or impaired redox homeostasis cause oxidative stress to play a pathogenic role in inflammation and fibrosis [65–67], the role of ROS and their regulation in EoE remain elusive. For example, excessive ROS damage cellular components such as mitochondria, major cellular sources of ROS. Dysfunctional mitochondria further generate ROS [68]. ROS are scavenged by many cellular antioxidant enzymes (e.g. superoxide dismutase, catalase) and non-enzymatic scavengers (e.g. vitamin E and glutathione). The antioxidant-activating transcription factor NRF2 is suppressed in EoE [61]. Besides antioxidants, ROS trigger autophagy, an adaptive response that degrades intracellular components such as damaged dysfunctional mitochondria under oxidative stress [69]. Impaired autophagic flux contributes to hepatic and intestinal fibrosis, the latter found in Crohn’s disease [70, 71]. We have demonstrated autophagy-mediated redox regulation in EoE. Autophagy-related gene products including ATG7 and LC3 regulate the formation of autophagy vesicles (AV), and ATG7 is an independent tissue biomarker for EoE inflammation in pediatric patients [72]. AV accumulation was further demonstrated in esophageal epithelia of EoE patients and mice with EoE-like inflammation [64]. Pharmacological inhibition of autophagy flux by hydroxychloroquine (HCQ) in EoE-bearing mice resulted in exacerbated oxidative stress, BCH and eosinophil infiltrates [64], indicating a protective role for autophagy in EoE [64]. Damaged mitochondria may undergo mitochondria-targeted autophagy, termed mitophagy [73]. The E3 ubiquitin ligase Parkin protein (PARK2 gene product) recruits damaged mitochondria to autophagic machinery [73–76]. Impaired Parkin-mediated mitophagy may have a role in the pathogenesis of pulmonary fibrosis [77]. Therefore, it is plausible that epithelial redox homeostasis and mitochondria-targeted autophagy may limit EoE-related fibrosis.

### Mechanical environment and fibrosis

In addition to the chemical environment impacting the behavior of fibroblasts, the mechanical environment also stimulates fibroblast activation. Fibroblasts are mechanosensitive, thus they sense the stiffness of their environment and react to it. We have recently shown that primary esophageal fibroblasts, when cultured in a relatively soft environment (1–3 kPa), display quiescent features. However, when placed in a stiff environment, the fibroblasts display enhanced proliferation, contractility, cell spreading, and α-SMA expression [38] even in the absence of exogenous cytokine stimulation. Autocrine TGF-β signaling has been implicated as SMAD3 phosphorylation was induced in fibroblasts as a function of stiffness. Fibroblasts, therefore, can differentiate into activated myofibroblasts without an inflammatory milieu in the setting of a stiff environment suggesting that even in the setting of disease remission, fibroblasts may continue to perpetuate matrix remodeling in a stiff environment.

### Potential therapeutic targets for fibrosis in EoE

Compared to other human diseases featuring fibrosis, there is a substantially large knowledge gap in the biological processes and profibrotic signaling pathways involved in the pathogenesis of EoE-related fibrosis. Despite extensive gene expression profiling in biopsies from EoE patients [42, 78–80], there is no reliable tissue biomarker of EoE-related esophageal fibrotic stricture. Potential therapeutic targets for EoE-related fibrosis include the biological processes, signaling pathways, cytokines and factors regulating recruitment and activation of fibroblasts, production and deposition of collagen and other ECM components and tissue stiffness. To this end, neutralizing antibodies for TGF-β [81] and other cytokines can be utilized. Small molecule inhibitors may suppress pertinent signaling pathways. Immunosuppressants such as rapamycin may activate autophagy to decrease oxidative stress. Rapamycin has been shown to attenuate fibrosis in the kidney and the liver [82, 83]. β-aminopropionitrile (BAPN) may suppress LOX-mediated collagen crosslinking [84]. Preclinical in vivo testing can be done in emerging murine models of EoE [13, 85, 86] although characterization of lamina propria fibrosis remains limited in these models.

## Clinical evaluation of remodeling in EoE

EoE was first described in the early 90′s in a series of reports describing a population of patients with refractory reflux symptoms and difficulty swallowing who had increased esophageal epithelial eosinophilia [87, 88]. As more was learned about EoE, it was noted that there is an age dependent difference in presenting symptoms in EoE. Young children present with feeding disorders and vomiting while older children and adults present with dysphagia and food impaction [89]. Others have shown that duration of untreated disease and symptoms correlate with stricture formation and fibrostenosis [8, 90, 91]. Furthermore, fibrostenotic phenotype as defined as the presence of rings or stricture on esophagogastroduodenoscopy increases with age. For every 10 years gained in age, there is double the risk of having fibrostenotic disease [90]. Taken together, this data suggest that EoE is a progressive disease with unchecked chronic inflammation leading to increased stiffness and fibrosis. However, there have not been long-term prospective studies that follow patients through the maturation process from childhood into adulthood to confirm these observed trends. An alternative theory is that this is a disease comprising inflammatory and fibrostenotic types [92]. Longitudinal studies to evaluate the progression of disease and improved methods of detecting fibrosis are needed to better delineate the natural history.

### Measurements of fibrostenotic disease

The current methods of evaluating for fibrostenosis include: (1) visual endoscopic evaluation, (2) esophagogram, and (3) histologic evaluation [93]. Visual inspection by endoscopy can identify rings and more severe narrow caliber esophagi, however esophagogram may be more sensitive at detecting more subtle esophageal narrowing [94]. Histologic evaluation of the lamina propria- the current gold standard is limited by sampling. This is especially true in pediatric samples where approximately 50% have adequate lamina propria for interpretation defined this as > 35 µm of thickness without crush effect [95]. Adult studies fare only slightly better with 61% with adequate lamina propria reported [96]. Taken together, this suggests that we are unable to assess fibrotic tissue remodeling in half of the EoE patients.

More recently, use of the endoscopic Endoluminal Functional Lumen Imaging probe (EndoFLIP) has provided information that the EoE esophagus is less distensible than the normal esophagus [49, 95, 97, 98]. Adult studies have shown that in EoE, patients with a history of food impaction have decreased distensibility and the distensibility is predictive of future food impaction [49, 98]. While all natural history studies show that fibrostenosis is less common in pediatrics, the pediatric esophagus in EoE is less distensible than non-EoE when controlling for age [95]. Importantly, decreased esophageal distensibility has been associated with the presence of lamina propria fibrosis in pediatric active EoE patients [95]. Moreover, pediatric patients with active EoE (> 15 eos per hpf) displayed decreased esophageal distensibility with a history of dysphagia or food impaction [95]. While the EndoFLIP is used largely as a research tool at this time, its use for clinical care is increasing. As more studies are done, this tool has significant promise as a method to detect subtle narrowing currently undetectable with other tools.

## Conclusions

An improved understanding of the microenvironment in the EoE esophagus has provided insights into the development of fibrosis. The mixed inflammatory infiltrate and cytokine redundancy have led to therapeutic challenges [99, 100]. Because of high rates of non-response with current therapeutic options [4] and high rates of fibrostenosis at diagnosis, consideration of novel therapeutic strategies directed at the activated myofibroblast and collagen deposition may improve patient symptoms more effectively than current strategies. Currently, we are treating fibrostenotic disease after it has occurred. Future research to detect sub-clinical fibrosis prior to the onset of narrowing and dysphagia will greatly improve patient outcomes and decrease the burden of disease.

## Abbreviations

α-SMA: α-Smooth muscle actin
AV: Autophagy vesicles
BAPN: Β-aminopropionitrile
BCH: Basal cell hyperplasia
CTGF: Connective tissue growth factor
ECM: Extracellular matrix
EMT: Epithelial-mesenchymal transition
EndoFLIP: Endoluminal functional lumen imaging probe
EoE: Eosinophilic esophagitis
HCQ: Hydroxychloroquine
IGFBP: Insulin-like growth factor binding protein
IL: Interleukin
IL-13R: IL-13 receptor
LOX: Lysyl oxidase
MMP: Matrix metalloproteinase
PAI-1: Plasminogen activator inhibitor 1
PPI: Proton pump inhibitor
ROS: Reactive oxygen species
TGF-β: Transforming growth factor-β
T_h_2: T helper-type 2
TNF-α: Tumor necrosis factor-α
TSLP: Thymic stromal lymphopoietin

## References

[1] Aceves SS (2014). Remodeling and fibrosis in chronic eosinophil inflammation. Dig Dis.

[2] Cheng E, Souza RF, Spechler SJ (2012). Tissue remodeling in eosinophilic esophagitis. Am J Physiol Gastrointest Liver Physiol.

[3] Cianferoni A, Spergel JM, Muir A (2015). Recent advances in the pathological understanding of eosinophilic esophagitis. Expert Rev Gastroenterol Hepatol..

[4] Dellon ES. Management of refractory eosinophilic oesophagitis. Nat Rev Gastroenterol Hepatol. 2017. 10.1038/nrgastro.2017.56 PMC8018607 28536485

[5] Furuta GT, Katzka DA (2015). Eosinophilic esophagitis. N Engl J Med.

[6] Merves J, Muir A, Modayur Chandramouleeswaran P, Cianferoni A, Wang ML, Spergel JM (2014). Eosinophilic esophagitis. Ann Allergy Asthma Immunol.

[7] Eluri S, Runge TM, Cotton CC (2016). The extremely narrow-caliber esophagus is a treatment-resistant subphenotype of eosinophilic esophagitis. Gastrointest Endosc.

[8] Singla MB, Chehade M, Brizuela D (2015). Early comparison of inflammatory vs. fibrostenotic phenotype in eosinophilic esophagitis in a multicenter longitudinal study. Clin Transl Gastroenterol..

[9] Dellon ES (2012). Eosinophilic esophagitis: diagnostic tests and criteria. Curr Opin Gastroenterol..

[10] Ueki S, Konno Y, Takeda M (2016). Eosinophil extracellular trap cell death-derived DNA traps: their presence in secretions and functional attributes. J Allergy Clin Immunol.

[11] Akuthota P, Weller PF (2012). Eosinophils and disease pathogenesis. Semin Hematol.

[12] Aceves SS, Chen D, Newbury RO (2010). Mast cells infiltrate the esophageal smooth muscle in patients with eosinophilic esophagitis, express TGF-beta1, and increase esophageal smooth muscle contraction. J Allergy Clin Immunol.

[13] Noti M, Wojno ED, Kim BS (2013). Thymic stromal lymphopoietin-elicited basophil responses promote eosinophilic esophagitis. Nat Med.

[14] Sayej WN, Menoret A, Maharjan AS (2016). Characterizing the inflammatory response in esophageal mucosal biopsies in children with eosinophilic esophagitis. Clin Transl Immunology..

[15] Wynn TA (2007). Common and unique mechanisms regulate fibrosis in various fibroproliferative diseases. J Clin Invest..

[16] Bochaton-Piallat ML, Gabbiani G, Hinz B. The myofibroblast in wound healing and fibrosis: answered and unanswered questions. F1000Res. 2016;5. 10.12688/f1000research.8190.1 PMC4847562 27158462

[17] Hill DA, Spergel JM (2016). The immunologic mechanisms of eosinophilic esophagitis. Curr Allergy Asthma Rep..

[18] Rieder F, Nonevski I, Ma J (2014). T-helper 2 cytokines, transforming growth factor beta1, and eosinophil products induce fibrogenesis and alter muscle motility in patients with eosinophilic esophagitis. Gastroenterology.

[19] Gieseck RL, Wilson MS, Wynn TA (2018). Type 2 immunity in tissue repair and fibrosis. Nat Rev Immunol.

[20] Chandramouleeswaran PM, Shen D, Lee AJ (2016). Preferential secretion of thymic stromal lymphopoietin (TSLP) by terminally differentiated esophageal epithelial cells: relevance to eosinophilic esophagitis (EoE). PLoS ONE.

[21] Rothenberg ME, Spergel JM, Sherrill JD (2010). Common variants at 5q22 associate with pediatric eosinophilic esophagitis. Nat Genet.

[22] Travers J, Rochman M, Caldwell JM (2017). IL-33 is induced in undifferentiated, non-dividing esophageal epithelial cells in eosinophilic esophagitis. Sci Rep..

[23] Blanchard C, Mingler MK, McBride M (2008). Periostin facilitates eosinophil tissue infiltration in allergic lung and esophageal responses. Mucosal Immunol.

[24] Blanchard C, Stucke EM, Burwinkel K (2010). Coordinate interaction between IL-13 and epithelial differentiation cluster genes in eosinophilic esophagitis. J Immunol..

[25] Atasoy U, Curry SL, Lopez de Silanes I (2003). Regulation of eotaxin gene expression by TNF-alpha and IL-4 through mRNA stabilization: involvement of the RNA-binding protein HuR. J Immunol..

[26] Cheng E, Zhang X, Huo X (2013). Omeprazole blocks eotaxin-3 expression by oesophageal squamous cells from patients with eosinophilic oesophagitis and GORD. Gut.

[27] Collins PD, Marleau S, Griffiths-Johnson DA (1995). Cooperation between interleukin-5 and the chemokine eotaxin to induce eosinophil accumulation in vivo. J Exp Med.

[28] Blanchard C, Mishra A, Saito-Akei H (2005). Inhibition of human interleukin-13-induced respiratory and oesophageal inflammation by anti-human-interleukin-13 antibody (CAT-354). Clin Exp Allergy: J Br Soc Allergy Clin Immunol..

[29] Mishra A, Rothenberg ME (2003). Intratracheal IL-13 induces eosinophilic esophagitis by an IL-5, eotaxin-1, and STAT6-dependent mechanism. Gastroenterology.

[30] Cheng E, Zhang X, Wilson KS (2016). JAK-STAT6 pathway inhibitors block eotaxin-3 secretion by epithelial cells and fibroblasts from esophageal eosinophilia patients: promising agents to improve inflammation and prevent fibrosis in EoE. PLoS ONE.

[31] Cho JY, Doshi A, Rosenthal P (2014). Smad3-deficient mice have reduced esophageal fibrosis and angiogenesis in a model of egg-induced eosinophilic esophagitis. J Pediatr Gastroenterol Nutr.

[32] Muir AB, Lim DM, Benitez AJ (2013). Esophageal epithelial and mesenchymal cross-talk leads to features of epithelial to mesenchymal transition in vitro. Exp Cell Res.

[33] Gomes I, Mathur SK, Espenshade BM (2005). Eosinophil-fibroblast interactions induce fibroblast IL-6 secretion and extracellular matrix gene expression: implications in fibrogenesis. J Allergy Clin Immunol..

[34] Murray LA, Zhang H, Oak SR (2014). Targeting interleukin-13 with tralokinumab attenuates lung fibrosis and epithelial damage in a humanized SCID idiopathic pulmonary fibrosis model. Am J Respir Cell Mol Biol.

[35] Zuo L, Fulkerson PC, Finkelman FD (2010). IL-13 induces esophageal remodeling and gene expression by an eosinophil-independent, IL-13R alpha 2-inhibited pathway. J Immunol..

[36] Singh B, Kasam RK, Sontake V (2017). Repetitive intradermal bleomycin injections evoke T-helper cell 2 cytokine-driven pulmonary fibrosis. Am J Physiol Lung Cell Mol Physiol.

[37] Aceves SS, Newbury RO, Dohil R (2007). Esophageal remodeling in pediatric eosinophilic esophagitis. J Allergy Clin Immunol..

[38] Muir AB, Dods K, Henry SJ (2016). Eosinophilic esophagitis-associated chemical and mechanical microenvironment shapes esophageal fibroblast behavior. J Pediatric Gastroenterol Nutr..

[39] Beppu L, Yang T, Luk M (2015). MMPs-2 and -14 are elevated in eosinophilic esophagitis and reduced following topical corticosteroid therapy. J Pediatr Gastroenterol Nutr.

[40] Rawson R, Yang T, Newbury RO (2016). TGF-beta1-induced PAI-1 contributes to a profibrotic network in patients with eosinophilic esophagitis. J Allergy Clin Immunol..

[41] Sherrill JD, Kc K, Wu D (2014). Desmoglein-1 regulates esophageal epithelial barrier function and immune responses in eosinophilic esophagitis. Mucosal Immunol.

[42] Wen T, Stucke EM, Grotjan TM (2013). Molecular diagnosis of eosinophilic esophagitis by gene expression profiling. Gastroenterology.

[43] Naik PK, Bozyk PD, Bentley JK (2012). Periostin promotes fibrosis and predicts progression in patients with idiopathic pulmonary fibrosis. Am J Physiol Lung Cell Mol Physiol.

[44] O’Dwyer DN, Moore BB (2017). The role of periostin in lung fibrosis and airway remodeling. Cell Mol Life Sci.

[45] Kaviratne M, Hesse M, Leusink M (2004). IL-13 activates a mechanism of tissue fibrosis that is completely TGF-beta independent. J Immunol..

[46] Mori T, Kawara S, Shinozaki M (1999). Role and interaction of connective tissue growth factor with transforming growth factor-beta in persistent fibrosis: a mouse fibrosis model. J Cell Physiol.

[47] Flynn RS, Mahavadi S, Murthy KS (2011). Endogenous IGFBP-3 regulates excess collagen expression in intestinal smooth muscle cells of Crohn’s disease strictures. Inflamm Bowel Dis.

[48] Trackman PC (2016). Lysyl oxidase isoforms and potential therapeutic opportunities for fibrosis and cancer. Expert Opin Ther Targets..

[49] Nicodeme F, Hirano I, Chen J (2013). Esophageal distensibility as a measure of disease severity in patients with eosinophilic esophagitis. Clin Gastroenterol Hepatol.

[50] Simon D, Radonjic-Hosli S, Straumann A (2015). Active eosinophilic esophagitis is characterized by epithelial barrier defects and eosinophil extracellular trap formation. Allergy.

[51] Rochman M, Travers J, Miracle CE, et al. Profound loss of esophageal tissue differentiation in patients with eosinophilic esophagitis. J Allergy Clin Immunol. 2017. 10.1016/j.jaci.2016.11.042 PMC5513800 28104354

[52] Collins MH, Martin LJ, Alexander ES (2017). Newly developed and validated eosinophilic esophagitis histology scoring system and evidence that it outperforms peak eosinophil count for disease diagnosis and monitoring. Dis Esophagus.

[53] Omori-Miyake M, Yamashita M, Tsunemi Y (2014). In vitro assessment of IL-4- or IL-13-mediated changes in the structural components of keratinocytes in mice and humans. J Invest Dermatol..

[54] Kagalwalla AF, Akhtar N, Woodruff SA (2012). Eosinophilic esophagitis: epithelial mesenchymal transition contributes to esophageal remodeling and reverses with treatment. J Allergy Clin Immunol.

[55] Ohashi S, Natsuizaka M, Wong GS (2010). Epidermal growth factor receptor and mutant p53 expand an esophageal cellular subpopulation capable of epithelial-to-mesenchymal transition through ZEB transcription factors. Cancer Res.

[56] Muir AB, Dods K, Noah Y (2015). Esophageal epithelial cells acquire functional characteristics of activated myofibroblasts after undergoing an epithelial to mesenchymal transition. Exp Cell Res.

[57] Ohashi S, Natsuizaka M, Naganuma S (2011). A NOTCH3-mediated squamous cell differentiation program limits expansion of EMT-competent cells that express the ZEB transcription factors. Cancer Res.

[58] Schoepfer A, Safroneeva E, Straumann A (2016). Eosinophilic esophagitis: impact of latest insights into pathophysiology on therapeutic strategies. Dig Dis.

[59] Sena LA, Chandel NS (2012). Physiological roles of mitochondrial reactive oxygen species. Mol Cell.

[60] Thannickal VJ, Fanburg BL (2000). Reactive oxygen species in cell signaling. Am J Physiol Lung Cell Mol Physiol.

[61] Jiang M, Ku WY, Zhou Z (2015). BMP-driven NRF2 activation in esophageal basal cell differentiation and eosinophilic esophagitis. J Clin Investig..

[62] Kinugasa H, Whelan KA, Tanaka K (2015). Mitochondrial SOD2 regulates epithelial-mesenchymal transition and cell populations defined by differential CD44 expression. Oncogene.

[63] Moqbel R, Lacy P (2000). Molecular mechanisms in eosinophil activation. Chem Immunol.

[64] Whelan KA, Merves JF, Giroux V (2017). Autophagy mediates epithelial cytoprotection in eosinophilic oesophagitis. Gut.

[65] Cheresh P, Kim SJ, Tulasiram S (2013). Oxidative stress and pulmonary fibrosis. Biochim Biophys Acta.

[66] Richter K, Kietzmann T (2016). Reactive oxygen species and fibrosis: further evidence of a significant liaison. Cell Tissue Res.

[67] Mittal M, Siddiqui MR, Tran K (2014). Reactive oxygen species in inflammation and tissue injury. Antioxid Redox Signal.

[68] Murphy MP (2009). How mitochondria produce reactive oxygen species. Biochem J..

[69] Mizushima N (2007). Autophagy: process and function. Genes Dev.

[70] Seki E, Brenner DA (2015). Recent advancement of molecular mechanisms of liver fibrosis. J Hepatobiliary Pancreat Sci..

[71] Nguyen HT, Lapaquette P, Bringer MA (2013). Autophagy and Crohn’s disease. J Innate Immun..

[72] Merves JF, Whelan KA, Benitez AJ (2016). ATG7 gene expression as a novel tissue biomarker in eosinophilic esophagitis. Am J Gastroenterol.

[73] Youle RJ, Narendra DP (2011). Mechanisms of mitophagy. Nat Rev Mol Cell Biol.

[74] Ding WX, Yin XM (2012). Mitophagy: mechanisms, pathophysiological roles, and analysis. Biol Chem.

[75] Ashrafi G, Schwarz TL (2013). The pathways of mitophagy for quality control and clearance of mitochondria. Cell Death Diff..

[76] Geisler S, Holmstrom KM, Skujat D (2010). PINK1/Parkin-mediated mitophagy is dependent on VDAC1 and p62/SQSTM1. Nat Cell Biol.

[77] Kobayashi K, Araya J, Minagawa S (2016). Involvement of PARK2-mediated mitophagy in idiopathic pulmonary fibrosis pathogenesis. J Immunol..

[78] Blanchard C, Wang N, Stringer KF (2006). Eotaxin-3 and a uniquely conserved gene-expression profile in eosinophilic esophagitis. J Clin Invest..

[79] Sherrill JD, Kiran KC, Blanchard C (2014). Analysis and expansion of the eosinophilic esophagitis transcriptome by RNA sequencing. Genes Immun.

[80] Shoda T, Wen T, Aceves SS (2018). Eosinophilic oesophagitis endotype classification by molecular, clinical, and histopathological analyses: a cross-sectional study. Lancet Gastroenterol Hepatol..

[81] Liang X, Schnaper HW, Matsusaka T (2016). Anti-TGF-beta antibody, 1D11, ameliorates glomerular fibrosis in mouse models after the onset of proteinuria. PLoS ONE.

[82] Liu CF, Liu H, Fang Y (2014). Rapamycin reduces renal hypoxia, interstitial inflammation and fibrosis in a rat model of unilateral ureteral obstruction. Clin Invest Med.

[83] Wang W, Yan J, Wang H (2014). Rapamycin ameliorates inflammation and fibrosis in the early phase of cirrhotic portal hypertension in rats through inhibition of mTORC1 but not mTORC2. PLoS ONE.

[84] Liu SB, Ikenaga N, Peng ZW (2016). Lysyl oxidase activity contributes to collagen stabilization during liver fibrosis progression and limits spontaneous fibrosis reversal in mice. FASEB J..

[85] Mishra A, Wang M, Pemmaraju VR (2008). Esophageal remodeling develops as a consequence of tissue specific IL-5-induced eosinophilia. Gastroenterology.

[86] Masterson JC, McNamee EN, Hosford L (2014). Local hypersensitivity reaction in transgenic mice with squamous epithelial IL-5 overexpression provides a novel model of eosinophilic oesophagitis. Gut.

[87] Kelly KJ, Lazenby AJ, Rowe PC (1995). Eosinophilic esophagitis attributed to gastroesophageal reflux: improvement with an amino acid-based formula. Gastroenterology.

[88] Attwood SE, Smyrk TC, Demeester TR (1993). Esophageal eosinophilia with dysphagia. A distinct clinicopathologic syndrome. Dig Dis Sci.

[89] Falk GW (2014). Clinical presentation of eosinophilic esophagitis in adults. Gastroenterol Clin North Am.

[90] Dellon ES, Kim HP, Sperry SL (2014). A phenotypic analysis shows that eosinophilic esophagitis is a progressive fibrostenotic disease. Gastrointest Endosc.

[91] Schoepfer AM, Safroneeva E, Bussmann C (2013). Delay in diagnosis of eosinophilic esophagitis increases risk for stricture formation in a time-dependent manner. Gastroenterology.

[92] Atkins D, Furuta GT, Liacouras CA (2017). Eosinophilic esophagitis phenotypes: ready for prime time?. Pediatr Allergy Immunol.

[93] Muir AB, Merves J, Liacouras CA (2016). Role of endoscopy in diagnosis and management of pediatric eosinophilic esophagitis. Gastrointest Endosc Clin N Am.

[94] Menard-Katcher C, Swerdlow MP, Mehta P (2015). Contribution of esophagram to the evaluation of complicated pediatric eosinophilic esophagitis. J Pediatr Gastroenterol Nutr.

[95] Menard-Katcher C, Benitez AJ, Pan Z (2017). Influence of age and eosinophilic esophagitis on esophageal distensibility in a pediatric cohort. Am J Gastroenterol.

[96] Safroneeva E, Straumann A, Coslovsky M (2016). Symptoms have modest accuracy in detecting endoscopic and histologic remission in adults with eosinophilic esophagitis. Gastroenterology.

[97] Carlson DA (2017). Editorial: widening the use of the functional lumen imaging probe to kids with eosinophilic esophagitis: esophageal narrowing is not just an adult problem. Am J Gastroenterol.

[98] Carlson DA, Lin Z, Hirano I (2016). Evaluation of esophageal distensibility in eosinophilic esophagitis: an update and comparison of functional lumen imaging probe analytic methods. Neurogastroenterol Motil.

[99] Spergel JM, Rothenberg ME, Collins MH (2012). Reslizumab in children and adolescents with eosinophilic esophagitis: results of a double-blind, randomized, placebo-controlled trial. J Allergy Clin Immunol..

[100] Rocha R, Vitor AB, Trindade E (2011). Omalizumab in the treatment of eosinophilic esophagitis and food allergy. Eur J Pediatr.

